# A systematic review and meta-analysis of the effects of green tea extracts and polyphenols in female hormone-dependent cancers for benefit-risk evaluation

**DOI:** 10.3389/fonc.2025.1579470

**Published:** 2025-09-22

**Authors:** Jin-jing He, Yan-fang Zhang, Ze-huang He, Lei Zheng

**Affiliations:** ^1^ Department of Operating Room, Zhejiang Hospital, Hangzhou, Zhejiang, China; ^2^ Department of Nephrology, The 903rd Hospital of The Chinese People’s Liberation Army, Hangzhou, Zhejiang, China; ^3^ Department of Vascular Surgery, Zhejiang Hospital, Hangzhou, Zhejiang, China; ^4^ Department of Breast Surgery, Zhejiang Hospital, Hangzhou, Zhejiang, China

**Keywords:** green tea extract, green tea polyphenols, breast cancer, ovarian cancer, uterine cancer, meta-analysis

## Abstract

Female hormone-dependent cancers rely on estrogen for growth and include breast, uterine and ovarian cancers. Although preclinical studies indicate that green tea extracts and polyphenols derived from green tea exhibit anti-tumor effects without mimicking estrogen like phytoestrogens, clinical evidence remains scarce. To explore the potential of green tea products in inhibiting these cancers, we conducted a meta-analysis of preclinical data. We evaluated the effects of green tea extract (GTE), green tea polyphenol-enriched product (GTP), and epigallocatechin gallate (EGCG) on tumor growth indices in mouse and rat models of breast, ovarian, and uterine cancers. A comprehensive search of PubMed, Web of Science, and Google Scholar (1998–2024) identified 20 studies for inclusion. Pooled analysis showed significant reductions in tumor volume (Hedge’s g = -2.332, 95% CI = -3.067 to -1.596, p = 0.000) and tumor weight (Hedge’s g = -2.105, 95% CI = -2.746 to -1.463, p = 0.000). Subgroup analysis revealed that GTE and EGCG reduced breast and ovarian tumors, while EGCG had no significant impact on uterine cancer. Significant heterogeneity was observed across studies. No consistent adverse effects were reported in the included studies, though liver function parameters were not assessed. These findings highlight the necessity for targeted clinical trials to assess the distinct benefits of each tea-based product for various cancer types.

## Introduction

Female hormone-dependent cancers, particularly breast, ovarian, and endometrial cancers, remain a significant health challenge worldwide. Breast cancer, in particular, is the most prevalent malignancy among women, accounting for approximately 25% of all female cancers globally (1). These cancers are largely driven by hormonal factors, such as estrogen and progesterone, which regulate tumor growth, proliferation, and survival. Although hormone therapies, such as selective estrogen receptor modulators (SERMs) and aromatase inhibitors, are effective treatment options, issues like drug resistance, recurrence, and adverse effects highlight the need for alternative or adjunctive therapeutic strategies (2).

In recent years, natural products and dietary supplements have gained attention for their role in cancer prevention and therapy (3). Tea (Camellia sinensis), particularly green tea, is notable for its high polyphenol content, especially catechins like epigallocatechin gallate (EGCG), a major catechin constituent (4) known for its antioxidant, anti-inflammatory, and anti-cancer properties (5). Green tea, unfermented and high in catechins, has been the focus of numerous studies, with its extracts standardized with well-established bioactive profile. As a result, green tea extract (GTE), green tea polyphenols-enriched extract (GTP) and EGCG have shown significant anti-cancer potential in preclinical models, highlighting the therapeutic promise (6).

Several *in vitro* and animal studies have indicated that GTE, GTP and EGCG exert anti-carcinogenic effects by targeting multiple molecular pathways (7). These pathways include the inhibition of cancer cell proliferation, induction of apoptosis, suppression of angiogenesis, and modulation of hormone receptor signaling pathways (4). Notably, in hormone-dependent cancers, these compounds appear to interfere with estrogen receptor (ER) activity, reduce estrogen synthesis, and modulate the cell cycle, leading to reduced tumor growth and progression (8, 9). EGCG, the most studied tea catechin, has been shown to inhibit the growth of ER+ breast cancer cells and to enhance the effects of standard therapies such as tamoxifen (10). Similarly, GTPs have demonstrated the ability to modulate critical signaling pathways, including those involved in hormone synthesis and receptor signaling, in animal models of hormone-dependent cancers (11). These findings suggest that GTPs could be valuable adjuncts to conventional therapies, particularly in preventing or overcoming drug resistance. However, translating these preclinical results into clinical practice remains a challenge due to the lack of robust human studies.

Despite the current absence of clinical evidence, a comprehensive evaluation of robust preclinical studies can yield pivotal insights into the therapeutic potential of tea-based products in female hormone-dependent cancers, providing a compelling rationale for clinical oncologists to design and conduct translational trials that bridge the gap between laboratory findings and clinical practice, ultimately informing evidence-based decision-making and paving the way for innovative treatments. Towards this aim, this systematic review and meta-analysis evaluates the preclinical efficacy of green tea extracts, polyphenol-enriched formulations, and the most abundant purified flavonoid in female hormone-dependent cancers, assessing their therapeutic potential and informing future clinical investigations.

## Material and methods

### Search strategy

**Figure 1:**
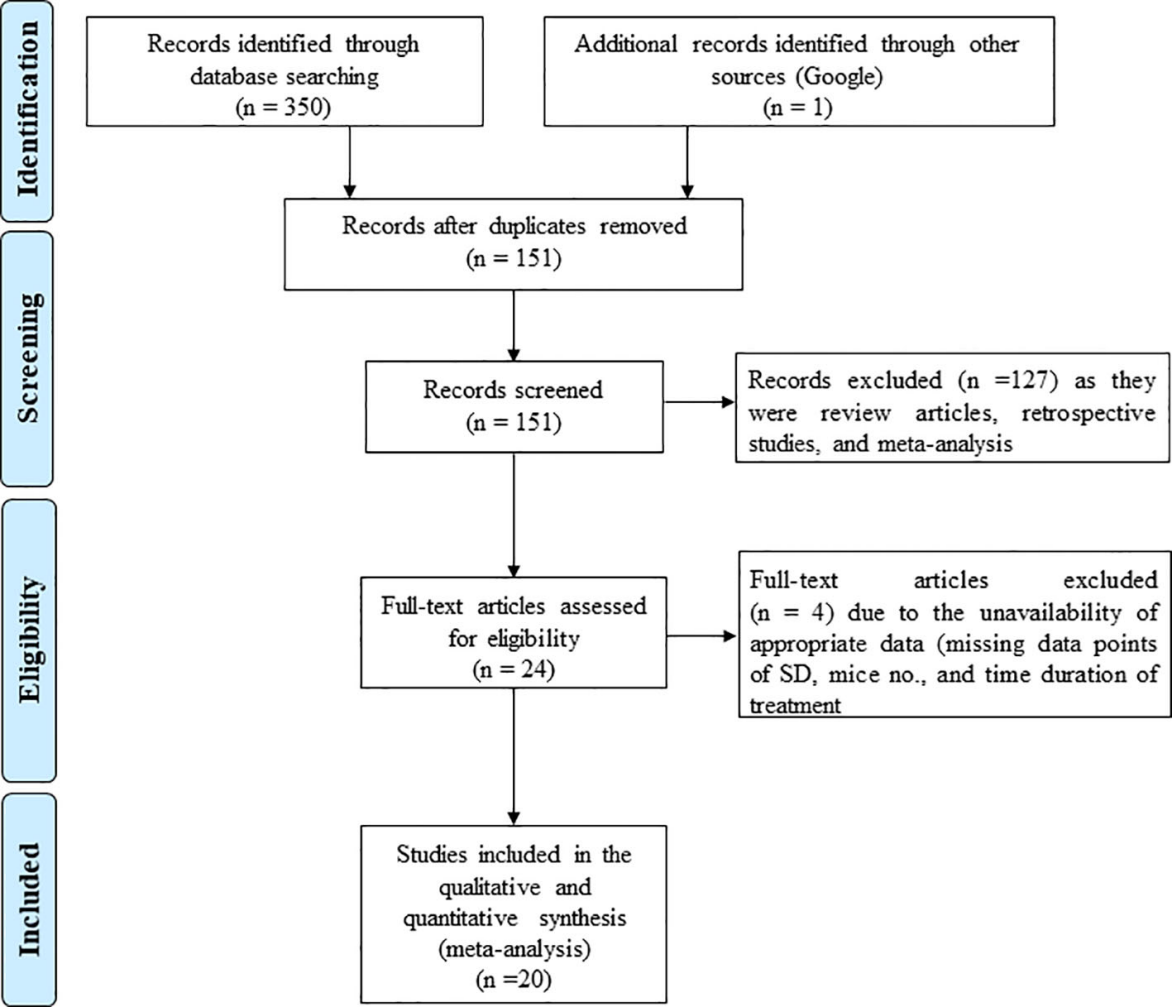
PRISMA flow diagram illustrating the process of identifying studies included for quantitative meta-analysis. The diagram details the number of records identified from databases (PubMed, Embase, Web of Science, Cochrane Library) with 1,234 and 12 records, respectively, leading to 20 included studies after screening and exclusions, as per PRISMA 2020 guidelines.

We conducted the literature search using three electronic databases, namely PubMed Medline, Web of Science, and Google Scholar to identify the studies that evaluated the effect of GTE, GTPs and individual components of tea polyphenols (TPs) including epicatechin, epigallocatechin, epicatechin gallate, EGCG, theaflavin-3,3’-digallate, thearubigins and theabrownins on tumor growth in experimental animal models by measuring tumor volume (TV) and tumor weight (TW) of female hormone-dependent cancers. The animal models used in the included studies are xenograft tumor models, ovariectomized xenograft tumor models, orthotopic tumor models, and patient derived xenograft (PDX) tumor in mice or rats. We searched different databases without specifying the timespan. The search terms included various components, including “Green tea extract, breast cancer, *in vivo*”; “Green tea polyphenols, breast cancer, *in vivo*”; “Catechin, breast cancer, *in vivo*”; “epicatechin, breast cancer, *in vivo*”; “epigallocatechin, breast cancer, *in vivo*”; “epicatechin gallate, breast cancer, *in vivo*”; “epigallocatechin gallate, breast cancer, *in vivo*; “theaflavin-3,3’-digallate, breast cancer, *in vivo*”; “thearubigins breast cancer, *in vivo*”; “theabrownins, breast cancer, *in vivo*”; “Green tea extract, ovarian cancer, *in vivo*”; “Green tea polyphenols, ovarian cancer, *in vivo*”; “Catechin, ovarian cancer, *in vivo*”; “epicatechin, ovarian cancer, *in vivo*”; “epigallocatechin, ovarian cancer, *in vivo*”; “epicatechin gallate, ovarian cancer, *in vivo*”; “epigallocatechin gallate, ovarian cancer, *in vivo*; “theaflavin-3,3’-digallate, ovarian cancer, *in vivo*”; “thearubigins ovarian cancer, *in vivo*”; “theabrownins, ovarian cancer, *in vivo*”; Green tea extract, uterine cancer, *in vivo*”; “Green tea polyphenols, uterine cancer, *in vivo*”; “Catechin, uterine cancer, *in vivo*”; “epicatechin, uterine cancer, *in vivo*”; “epigallocatechin, uterine cancer, *in vivo*”; “epicatechin gallate, uterine cancer, *in vivo*”; “epigallocatechin gallate, uterine cancer, *in vivo*; “theaflavin-3,3’-digallate, uterine cancer, *in vivo*”; “thearubigins uterine cancer, *in vivo*”; “theabrownins, uterine cancer, *in vivo*”. Moreover, we manually searched the references cited in the relevant articles. The literature search results are outlined in the Preferred Reporting Items for Systematic Reviews and Meta-Analyses (PRISMA) flow chart (**Figure 1**).

### Study selection (inclusion and exclusion criteria)

We established specific inclusion/exclusion criteria for the results from the literature search and screened them accordingly. Inclusion criteria were: (1) original and full-length articles; (2) studies where GTE, GTPs and individual components of GTPs including epicatechin, epigallocatechin, epicatechin gallate, and EGCG were administered; (3) studies where xenograft tumor models or ovariectomized xenograft tumor models or PDX tumor models were used; (4) studies using laboratory animals; and (5) articles were published in English. The exclusion criteria were: (1) review articles; (2) clinical reports and/or trials; (3) reports wherein the *in vitro* effect of tea catechin was studied; and (5) studies that failed to provide the required information. There were no restrictions regarding species, age, gender, duration of tumor induction, and administration of GTE, GTP, and EGCG. The screening process involved two stages: initial title and abstract screening by two investigators (J-JH, Y-FZ), followed by full-text review by three investigators (J-JH, Y-FZ, Z-HH), with disagreements resolved through discussion with the senior author (LZ).

### Data extraction

Three investigators (J-JH, Y-FZ, and Z-HH) independently screened the literature, resolving any disagreements through discussion with other authors. Data were numerically extracted from bar plots in each article using the WebPlotDigitilizer program and from the tables, then presented in a Microsoft Excel spreadsheet (Windows 10 edition; Microsoft Corporation, Lisbon, Portugal) to record species and strains, the number of animals/groups, model cell lines used for tumor induction, the regimen of administration of tea-based products, and mean value of tumor parameters (TV and TW) with standard deviation. Breast cancer cell lines were categorized by hormone receptor status (e.g., ER+/PR+ for MCF - 7, triple-negative for MDA-MB-231).

### Quantitative data analysis

Pooled data analysis utilized Comprehensive Meta-Analysis Software Version 2, with Hedge’s g selected as the ‘effect size’ metric. Heterogeneity across studies was assessed using Cochran’s Q test and heterogeneity index (I^2^). A significance threshold of p < 0.10 was applied due to the test’s sensitivity. Quantitative assessment of heterogeneity used the I^2^ scale: low (<25%), moderate (50%), and high (>75%). The fixed effect or random effects model was chosen for computing the pooled effect size based on the level of heterogeneity. Sub-group analysis was conducted based on cancer type, hormone receptor status, and specific components of tea polyphenols.

### Sensitivity analysis

Sensitivity analysis was conducted by systematically excluding each study one at a time to assess its impact on the pooled effect size. This method was used to evaluate the robustness of the overall findings and determine the influence of individual studies on the meta-analysis results.

### Publication bias analysis

Publication bias was assessed both qualitatively and quantitatively. Qualitative evaluation was based on visual inspection of funnel plot asymmetry, while quantitative assessment was performed using Egger’s intercept test. In cases where publication bias was detected, Duval and Tweedie’s trim-and-fill method was applied to adjust the pooled estimates and inform the final conclusions.

## Results

### Study design and parameters measured

A total of 350 potential articles were identified from the databases PubMed, Embase, Web of Science, and Cochrane Library. Of these, 20 studies for TV and 16 for TW matched the inclusion criteria and were suitable for our meta-analysis, focusing on female hormone-dependent cancers, including breast, ovarian, and uterine cancers (12–31) and 16 for TW (13, 14, 17, 20, 22, 27–29, 31–38).

The literature review and study screening results are shown in the PRISMA flow diagram in **Figure 1**. Cancer models were created either by xenograft tumor implantation in rats/mice. GTE, GTP and EGCG were administered through different modes of delivery (drinking water, subcutaneous injection, intraperitoneal injection, intratumoral injection, intravenous injection, and infusion or oral gavage) and dosage forms as indicated in **Tables 1** and **2**. The sample size of the study ranged from 3 to 12, and the duration of treatment ranged from 2 weeks to 10 weeks. We performed a meta-analysis to analyze the effects GTE, GTP and EGCG on TV and TW. We used the random-effects model for making inferences due to significant heterogeneity across the studies unless stated otherwise. The pooled and subgroup analyses of all parameters, including TV and TW have been summarized in **Table 3**.

**Table 1:** Methodological characteristics of the studies included in the meta-analysis.

References	Gender, species, strain, age	Model cell line	Experiment				Outcome (TV mm^3^)					
			Dosage	Mode of treatment	Administration (treatment)	Duration of treatment	Mean 0	SD0	N0	Mean1	SD1	N1
Sartippour et al. (12)	Female SCID mice, 8 – 10 weeks	MDA-M 231	0.62mg/ml	Therapeutic	Drinking water (GTE)	35 days	1310.79	861.44	12	858.79	680.37	12
Sartippour et al. (12)	Female SCID mice, 8 – 10 weeks	MDA-M 231	1.25mg/ml	Therapeutic	Drinking water (GTE)	35 days	1310.79	861.44	12	572.54	468.131	12
Sartippour et al. (12)	Female SCID mice, 8 – 10 weeks	MDA-M 231	2.5mg/ml	Therapeutic	Drinking water (GTE)	35 days	1310.79	861.44	12	131.34	224.683	12
Zhou et al. (13)	Female SCID mice, 5 – 8 weeks	MCF-7	1.5%	Preventive	Infusion (GTE)	56 days	15.80	0.1	12	7.02	0.20	12
Baliga et al. (14)	Female BALB/c mice, 6 – 7 weeks	4T1 (HRG)	0.2%	Preventive	Drinking water (GTP)	36 days	1976.90	176.63	10	1209.23	148.77	10
Baliga et al. (14)	Female BALB/c mice, 6 – 7 weeks	4T1 (HRG)	0.5%	Preventive	Drinking water (GTP)	36 days	1976.90	176.63	10	862.77	112.09	10
Baliga et al. (14)	Female BALB/c mice, 6 – 7 weeks	4T1 (LRG)	0.2%	Preventive	Drinking water (GTP)	60 days	994.756	88	10	506.10	81	10
Sartippour et al. (15)	Ovariectomized nude mice, 6 weeks	MCF-7	2.5mg/ml	Therapeutic	Drinking water (GTE)	64 days	622.2	1524.0	6	341.1	119.53	6
Spinella et al. (16)	Female athymic (nu+/nu+) mice, 4 – 6 weeks,	HEY cells	12.4mg/ml	Therapeutic	Drinking water (GTE)	60 days	7260	1249	10	3208	908	10
Thangapazham et al. (19)	Female athymic nude mice, 5 weeks	MDA-MB-231	1%	Preventive	Drinking water (GTP)	70 days	31.649	100.08	10	12.540	9.0963	10
Thangapazham et al. (19)	Female athymic nude mice, 5 weeks	MDA-MB-231	1mg/animal	Preventive	Drinking water (EGCG)	70 days	31.661	109.67	10	17.703	10.101	10
Landis-Piwowar et al. (18)	Female athymic nude mice, 5 weeks	MDA-MB-231	50mg/kg	Therapeutic	Daily s.c. injection (EGCG)	31 days	1582	29	4	1223	21	4
Kaur et al. (17)	T antigen transgenic mice	MDA-MB-468	0.01%	Therapeutic	Drinking water (GTE)	130 days	369.3133	355.31	14	38.572	35.440	14
Scandlyn et al. (20)	Female CD1 athymic nude mice, 5 – 6 weeks	MDA-MB-231	25mg/kg	Therapeutic	Intraperitoneally (EGCG)	70 days	590.16	1866.2	10	409	306.42	10
Zhang et al. (22)	Female athymic nude mice, 5 – 6 weeks	ELT3 cells	1.25mg/day	Therapeutic	Drinking water (EGCG)	56 days	28	57	10	129	54	10
Luo et al. (21)	Female Balb/c mice, 6 – 7 weeks	4T1	30mg/kg	Therapeutic	Intraperitoneally (EGCG)	24 days	782.008	3028.7	15	709.28	417.19	15
Jang et al. (23)	Female BALB/c mice	4T1	10mg/kg	Therapeutic	Intraperitoneally (EGCG)	35 days	8124.1	14071	3	3570.13	1176.062498	3
Wang et al. (35)	Female BALB/c nude mice, 3 – 5 weeks	OVCAR3	20mg/kg	Therapeutic	Intraperitoneal injection (EGCG)	28 days	1773.02	104.43	6	1190.741	132.9239	8
Zhou et al. (25)	Female athymic nude mice	MCF-7	50mg/kg	Therapeutic	Intraperitoneal injection (EGCG)	24 days	1166.51	3688.8	10	965.85	442.71	10
Wang et al. (36)	Female athymic nude mice, 5 – 6 weeks	RL95–2	50mg/kg	Therapeutic	Oral gavage (EGCG)	35 days	489.084	1093.6	5	449.07	44.743	5
Wang et al. (36)	Female athymic nude mice, 5 – 6 weeks	AN3 CA	50mg/kg	Therapeutic	Oral gavage (EGCG)	21 days	1357.37	3035.1	5	1066.6	209.47	5
Lee et al. (30)	female NSG mice	Patient derived tumor	50mg/kg	Therapeutic	Subcutaneous injection (EGCG)	11 days	2.36444	4.7288	4	1.040	0.4275	4
Kazi et al. (27)	Nude mice	MDA-MB231	25mg/kg	Therapeutic	Intravenous (EGCG)	21 days	2617	115.6	5	981.3	55.8	5
Qin et al. (28)	Female BALB/c nude mice, 4 – 5 weeks	SKOV3	10mg/kg	Therapeutic	NG (EGCG)	21 days	909.787	59.936	7	701.59	72.554	7
Qin et al. (28)	Female BALB/c nude mice, 4 – 5 weeks	SKOV3	30mg/kg	Therapeutic	NG (EGCG)	21 days	909.787	59.936	7	526.51	42.58	7
Qin et al. (28)	Female BALB/c nude mice, 4 – 5 weeks	SKOV3	50mg/kg	Therapeutic	NG (EGCG)	21 days	909.787	59.936	7	324.62	23.65	7
Das et al. (29)	Female athymic nude mice, 6 – 8 week	MDA-M 231	100mg/kg	Therapeutic	Oral (EGCG)	21 days	365.493	161.22	4	120.00	49.465	4
Li et al. (31)	Nude mice, NG	A2780/DDP	50mg/kg	Therapeutic	Intraperitoneal injection (EGCG)	28 days	602.799	238.09	5	165.82	43.42	5
Qin et al. (28)	Female BALB/c nude mice, 4 – 5 weeks	SKOV3	50mg/kg	Therapeutic	NG (EGCG)	21 days	909.787	59.936	7	324.62	23.65	7
Das et al. (29)	Female athymic nude mice, 6 – 8 week	MDA-M 231	100mg/kg	Therapeutic	Oral (EGCG)	21 days	365.493	161.22	4	120.00	49.465	4
Li et al. (31)	Nude mice, NG	A2780/DDP	50mg/kg	Therapeutic	Intraperitoneal injection (EGCG)	28 days	602.799	238.09	5	165.82	43.42	5

Mean0, mean value in the control group (mm^3^ for TV, and gm for TW); Sd0, standard difference in the control group; N0, sample size in the control group; Mean1, mean in treatment group; Sd1, standard difference in treatment group; N1, sample size in treatment group; NR, non-reported; TV, tumor volume; GTE, Green tea extract and EGCG, epigallocatechin gallate, HRG, High risk group, LRG, Low risk group, BLC, biluochun, LJ, longjing, GGN, gougunao,WT, Anji white tea

**Table 2:** Methodological characteristics of the studies included in the meta-analysis.

References	Gender, species, strain, age	Model cell line	Experiment				Outcome type (TW in g)					
			Dosage	Mode of treatment	Administration (treatment)	Duration of treatment	Mean 0	SD0	N0	Mean1	SD1	N1
Kavanagh et al. (32)	Female SD rats, 4-week	DMBA	0.30%	Preventive	Drinking water (GTE)	84 days	8.3	6.9	15	2.5	4.5	15
Zhou et al. (13)	Female SCID mice, 5 – 8 weeks	MCF-7	1.50%	Preventive	Infusion (GTE)	56 days	1.59	0.38	12	0.69	0.358	12
Baliga et al. (14)	Female BALB/c mice, 6 – 7 week	4T1 (HRG)	0.2%	Preventive	Drinking water (GTP)	30 days	4.5	0.3	10	3.4	0.3	10
Baliga et al. (14)	Female BALB/c mice, 6 – 7 week	4T1 (HRG)	0.5%	Preventive	Drinking water (GTP)	30 days	4.5	0.3	10	2.1	0.3	10
Kaur et al. (17)	T antigen transgenic mice	MDA-MB-468	0.01%	Therapeutic	Drinking water (GTE)	130 days	1.93	1.85	16	1.44	0.53	16
Scandlyn et al. (20)	Female CD1 athymic nude mice, 5 – 6 weeks	MDA-MB-231	25 mg/kg	Therapeutic	Intraperitonial (EGCG)	70 days	0.45	0.08	10	0.29	0.4	10
Zhang et al. (22)	Female athymic nude mice, 5 – 6 weeks	ELT3 cells	1.25mg/day	Therapeutic	Drinking water (EGCG)	56 days	0.29	0.06	10	0.11	0.04	10
Luo et al. (33)	Female BALB/c mice, 6 – 8 weeks	4T1	0.6g/kg	Therapeutic	Oral (GTE)	28 days	1.76	0.474342	10	1.246	0.442719	10
Luo et al. (34)	Female BALB/c mice, 6 – 8 weeks	4T1	0.6g/kg	Therapeutic	Oral (GTE)	28 days	1.958	0.664078	10	1.23	0.373149	10
Wang et al. (35)	Female BALB/c nude mice, 3 – 5 weeks	OVCAR3	20mg/kg	Therapeutic	Intraperitoneal injection (EGCG)	28 days	1.460288	0.3857	6	0.992897	0.3399	8
Wang et al. (36)	Female athymic nude mice (nu/nu),5–6 weeks	RL95–2	50mg/kg	Therapeutic	Oral gavage (EGCG)	35 days	0.55	0.096151	5	0.58	0.111803	5
Wang et al. (36)	Female athymic nude mice (nu/nu),5–6 weeks	AN3 CA	50mg/kg	Therapeutic	Oral gavage (EGCG)	35 days	1.464794	0.536656	5	1.226807	0.402492	5

Mean0, mean value in the control group (mm^3^ for TV, and gm for TW); Sd0, standard difference in the control group; N0, sample size in the control group; Mean1, mean in treatment group; Sd1, standard difference in treatment group; N1, sample size in treatment group; NR, non-reported; TW, tumor weight, GTE, Green tea extract and EGCG, epigallocatechin gallate, HRG, High risk group, LRG, Low risk group, BLC, biluochun; LJ, longjing; GGN, gougunao; WT, Anji white tea

**Table 3:** Summary of the pooled data and subgroup analysis of various parameters of the study.

Parameter	Groups	Cancer type	Test of heterogeneity			Test Model	Types of association				Significance
		Female hormone-dependent cancers	Q	P	I^2^ (%)		Hedge’s g	Lower limit	Upper limit	P value	
TV	Control vs GTE, GTP and EGCG (Pooled group)	Breast cancer, Ovarian cancer Uterine Cancer	262.234	0.000	89.704	Random	-2.332	-3.067	-1.596	0.000	Significant
	Control vs GTE	Breast cancer, Ovarian cancer	64.434	0.000	90.688	Random	-1.766	-3.104	-0.429	0.010	Significant
	Control vs EGCG	Breast Cancer Ovarian cancer Uterine Cancer	127.408	0.000	87.442	Random	-2.061	-2.972	-1.150	0.000	Significant
	Control vs. GTE	Breast Cancer	52.654	90.504	0.000	Random	-1.073	-1.618	-0.528	0.000	Significant
	Control vs. GTP	Breast Cancer	56.700	94.709	0.000	Random	-4.281	-7.692	-0.869	0.014	Significant
	Control vs. EGCG	Breast Cancer	38.701	79.329	0.000	Random	-0.806	-1.688	0.077	0.074	Non-significant
	Control vs. EGCG	Ovarian Cancer	23.003	82.611	0.000	Random	-5.009	-7.251	-2.766	0.000	Significant
	Control vs. EGCG	Uterine Cancer	13.221	84.873	0.001	Random	-0.959	-2.658	0.740	0.269	Non-significant
TW	Control vs GTE, GTP and EGCG (Pooled group)	Breast cancer, Ovarian cancer, Uterine cancer	151.169	84.124	0.000	Random	-2.105	-2.748	-1.463	00.000	Significant
	Control vs. EGCG	Breast cancer, Ovarian cancer, Uterine cancer	91.527	85.797	0.000	Random	-2.885	-3.969	-1.800	0.000	Significant
	Control vs. GTE	Breast Cancer	14.013	42.909	0.081	Fixed	-0.873	-1.194	-0.552	0.000	Significant
	Control vs. EGCG	Breast Cancer	27.467	81.797	0.000	Random	-2.963	-4.530	-1.396	0.000	Significant
	Control vs. EGCG	Ovarian Cancer	38.453	89.598	0.000	Random	-4.703	-7.275	-2.132	0.000	Significant
	Control vs. EGCG	Uterine Cancer	17.767	88.743	0.000	Random	-3.742	-6.673	-0.811	0.012	Significant

### Effects of GTE, GTP and EGCG on tumor burdens of female-hormone dependent cancers

#### TV

**Figure 2:**
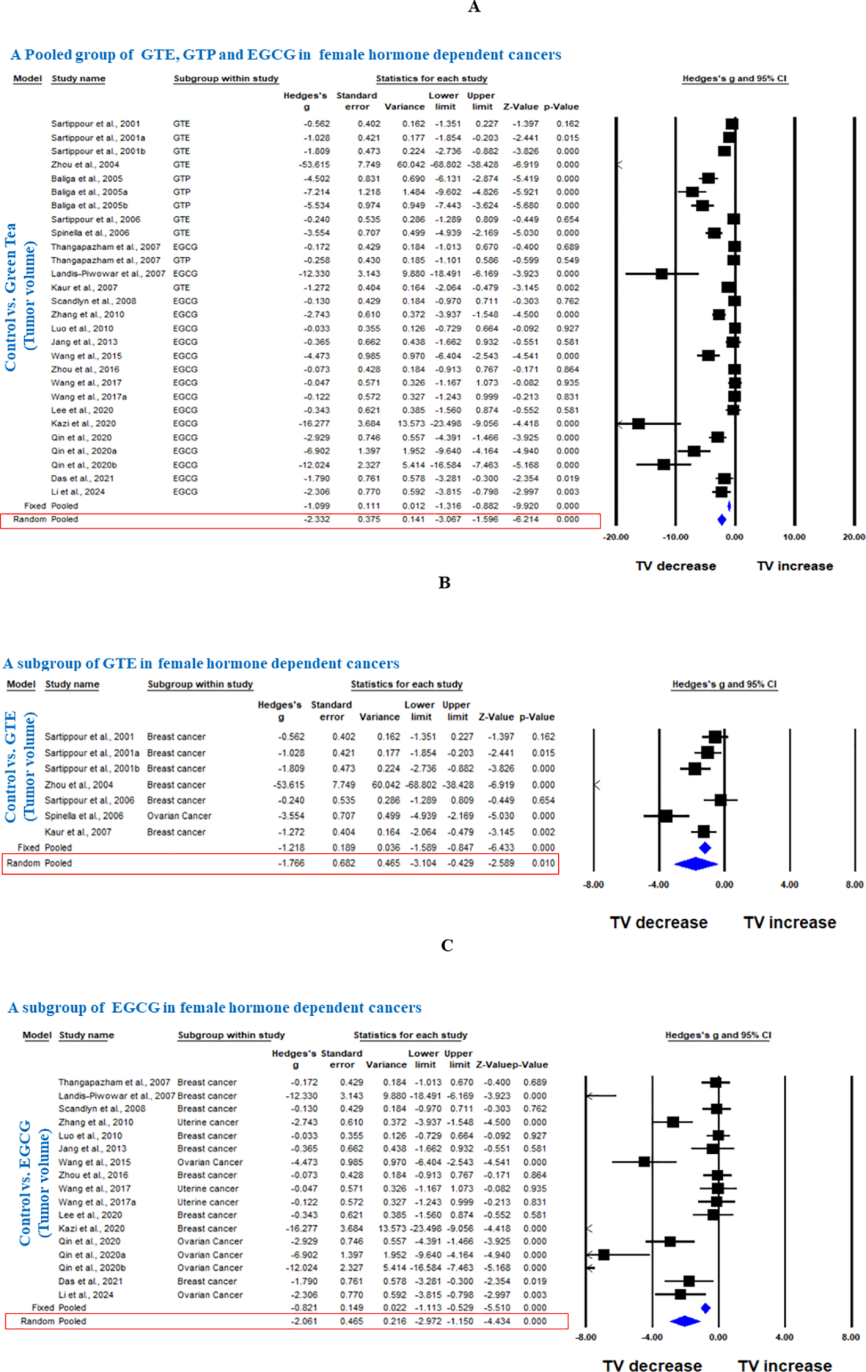
Green tea inhibits TV of female hormone-dependent cancers. **(A)** Forest plot of a pooled analysis of GTE, GTP and EGCG, **(B)** Forest plot of a subgroup analysis of GTE, and **(C)** Forest plot of a subgroup analysis of EGCG.

Twenty studies using xenograft models or an induced tumor model were included to investigate the effect of GTE, GTP and EGCG on TV in mice or rat models of three female hormone-dependent cancers (breast-, ovarian-, and uterine cancers) as shown in **Table 1** **. A** total of 237 animals were in the intervention group, while 235 animals were in the control group. The pooled analysis was performed using a random-effect model, which showed a significant decrease in TV upon treatment of GTE, GTP and EGCG (Hedge’s g = -2.332, 95% CI = -3.067 to -1.596, p = 0.000) as shown in **Figure 2**. The funnel plots did not demonstrate apparent asymmetry for TV and the heterogeneity among studies was significant (p=0.000, I^2^ = 89.704%, Q = 262.234).

Next, we conducted a subgroup analysis of GTE and EGCG separately on TV of the three female hormone-dependent cancers. GTE significantly decreased the TV in breast and ovarian cancers (Hedge’s g = -1.766, 95% CI = -3.104 to -0.429, p = 0.010) (**Figure 2**). EGCG decreased the TV in all three cancers types (Hedge’s g = -2.061, 95% CI = -2.972 to -1.150, p = 0.000) (**Figure 2**). The funnel plots did not demonstrate apparent asymmetry for TV and the heterogeneity among studies was significant for both GTE (p=0.000, I^2^ = 90.688%, Q = 64.434) and EGCG (p=0.000, I^2^ = 87.422%, Q = 127.408).

**Figure 3:**
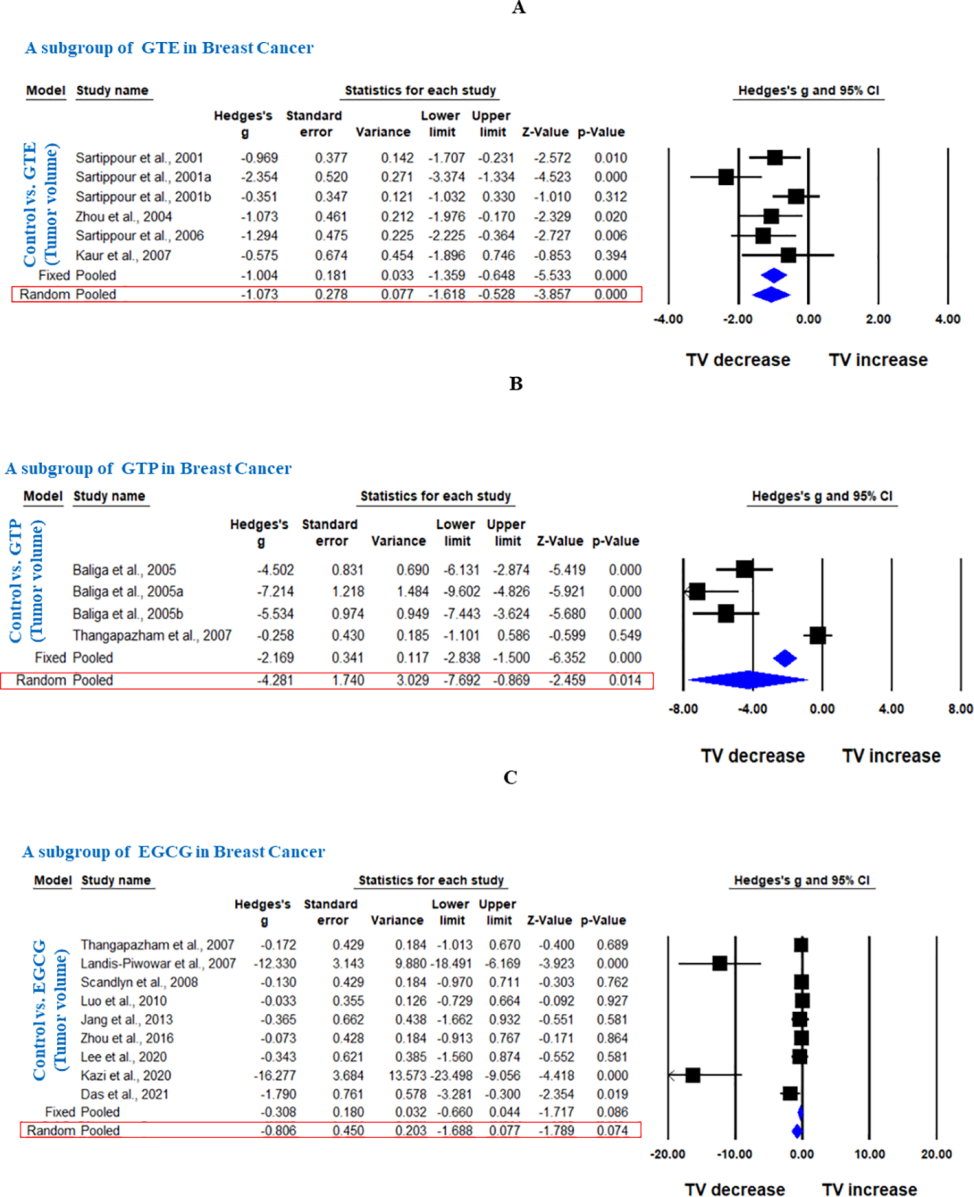
GTE, GTP, and EGCG inhibit TV of breast cancer. A subgroup analysis was shown in **(A)** Forest plot of GTE, **(B)** Forest plot of GTP, and **(C)** Forest plot of EGCG.

In another subgroup, we analyzed the effect of GTE, GTP, or EGCG on breast cancer. GTE significantly decreased the TV, however, there was significant heterogeneity among studies (Hedge’s g = -1.073, 95% CI = -1.618 to -0.528, p = 0.000, I^2^ = 90.504%, Q = 52.654, p=0.000) (**Figure 3**). GTP also significantly decreased the TV in breast cancer (Hedge’s g = -4.281, 95% CI = -7.692 to -0.869, p = 0.014) (**Figure 3**), however, EGCG did not have any significant effect (Hedge’s g = -0.806, 95% CI = -1.688 to 0.077, p = 0.074 (**Figure 3** **).** Subgroup analysis of GTP and EGCG individually to analyze the effect on TV in breast cancer also showed significant heterogeneity among studies (p= 0.000, I^2^ = 94.709%, Q = 56.7 for GTP and p= 0.000, I^2^ = 79.329%, Q = 38.701) and funnel plots did not demonstrate obvious asymmetry. Notably, studies using triple-negative MDA-MB-231 cells showed variable EGCG efficacy compared to hormone-responsive, MCF - 7 cells (20).

**Figure 4:**
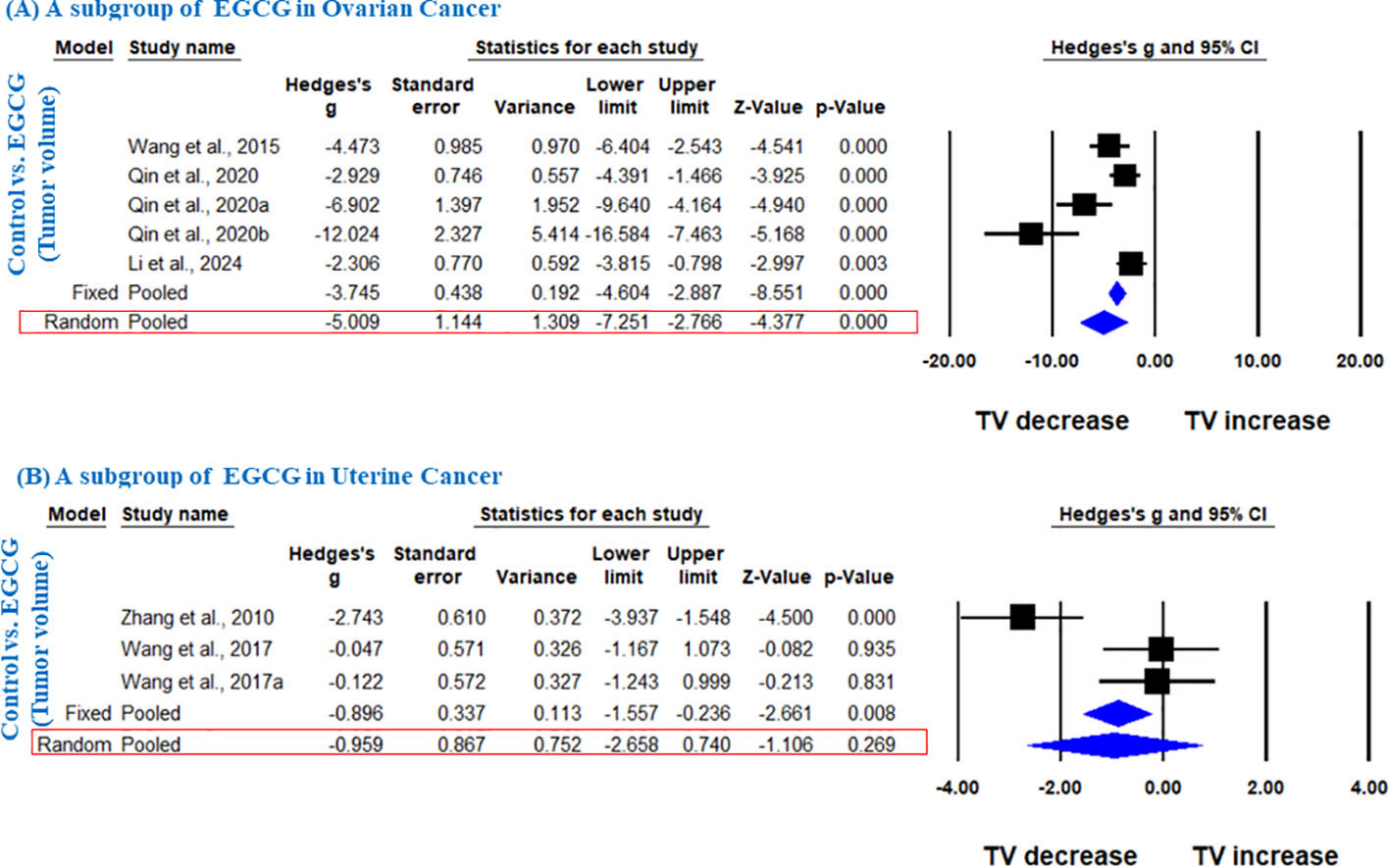
EGCG inhibit TV of ovarian and uterine cancer. A subgroup analysis was shown **(A)** Forest plot of EGCG in ovarian cancer, and **(B)** Forest plot of EGCG in uterine cancer.

Further, in another subgroup, we analyzed the effect of EGCG on ovarian and uterine cancer. EGCG significantly decreased the TV in ovarian cancer (Hedge’s g = -5.009, 95% CI = -7.251 to -2.766, p = 0.000, I^2^ = 82.611%, Q = 23.003, p=0.000) (**Figure 4**), however; it did not significantly decrease the TV in uterine cancer as shown in **Figure 4** (Hedge’s g = -0.959, 95% CI = -2.658 to 0.740, p = 0.269, I^2^ = 84.873%, Q = 13.221, p= 0.001).

#### TW

**Figure 5:**
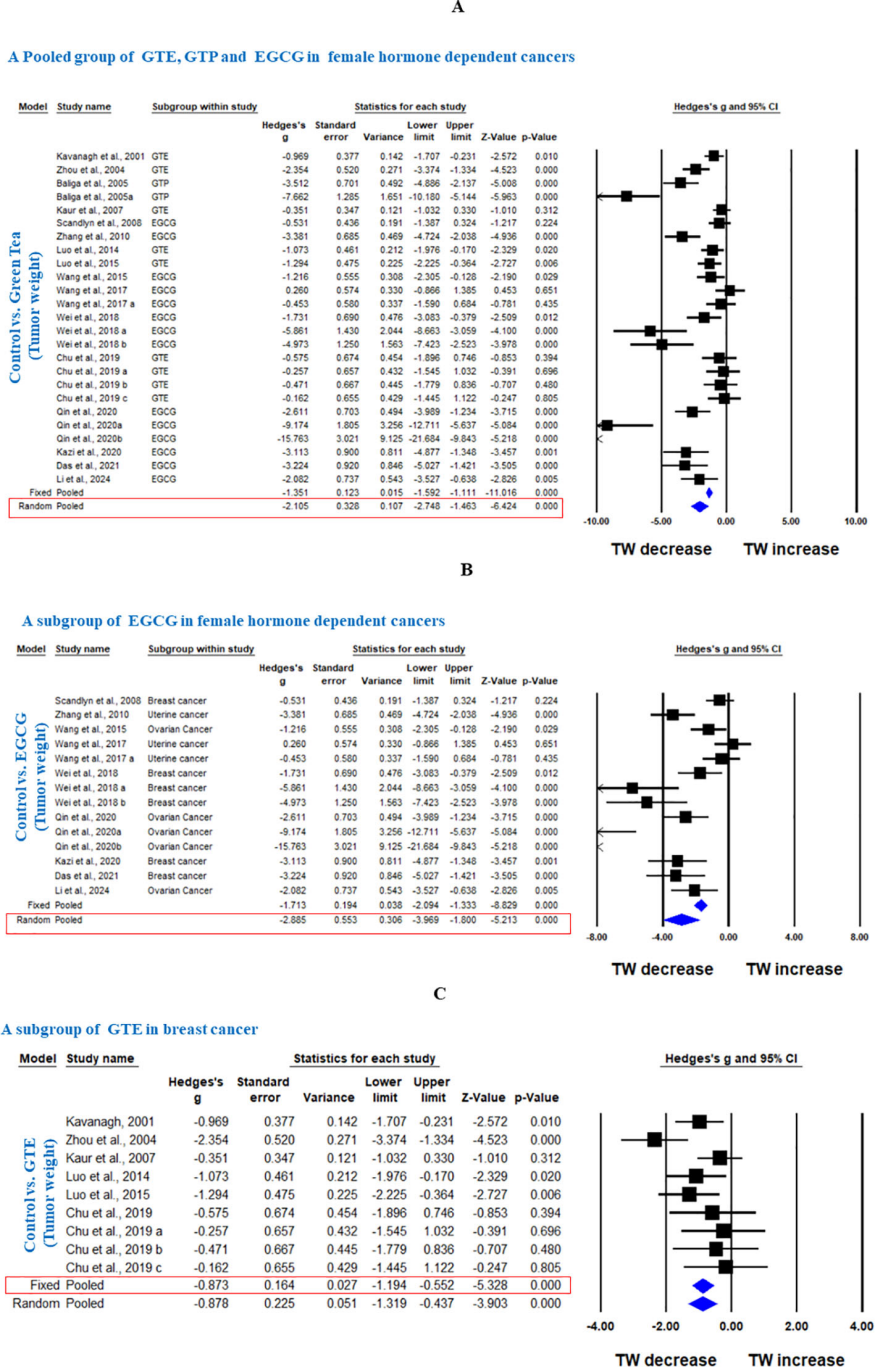
Green tea inhibits TW of female hormone-dependent cancers and breast cancer. **(A)** Forest plot of pooled analysis of GTE, GTP and EGCG on TW of female hormone-dependent cancers, **(B)** Forest plot of EGCG on TW of female hormone-dependent cancers, and **(C)** Forest plot of GTE on TW of breast cancer.

Sixteen studies using xenograft models or an induced tumor model were included to investigate the effect of GTE, GTP and EGCG on TW of three female hormone-dependent cancers as indicated in **Table 2**. The pooled analysis was performed using a random-effect model, which showed significant inhibition of TW upon treatment of GTE, GTP and EGCG in all three cancer types (Hedge’s g = -2.105, 95% CI = -2.746 to -1.463, p = 0.000) (**Figure 5**). The heterogeneity among studies was relatively high (p=0.000, I^2^ = 84.124%, Q = 151.169).

A subgroup analysis revealed that EGCG reduced TW across all three cancer types (Hedge’s g = -2.885, 95% CI = -3.969 to -1.800, p = 0.000, I^2^ = 85.797%, Q = 91.527, p=0.000) (**Figure 5**).

**Figure 6:**
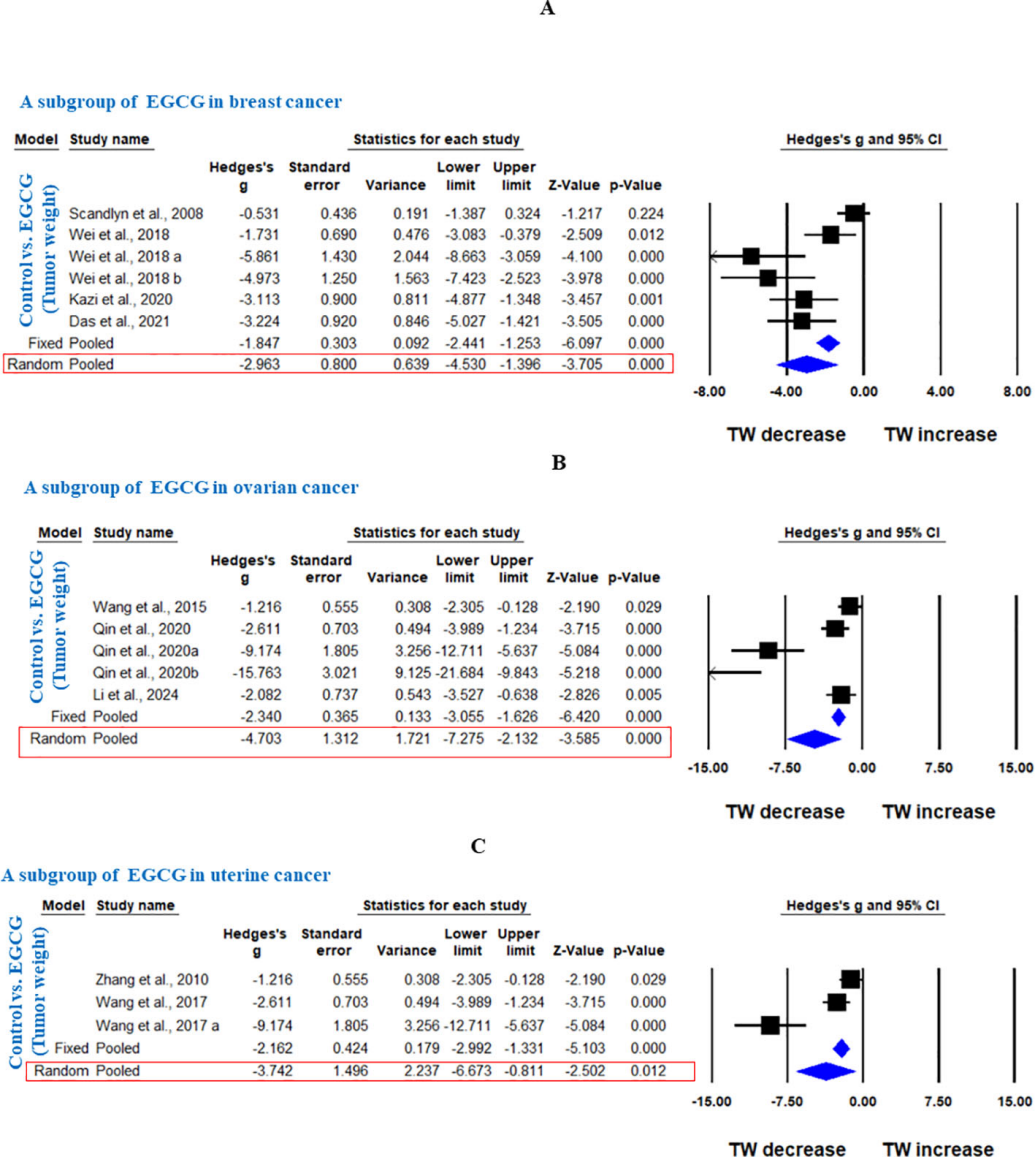
EGCG inhibits TW of breast cancer, ovarian cancer and uterine cancer. A subgroup analysis was shown **(A)** Forest plot of EGCG in breast cancer, **(B)** Forest plot of EGCG in ovarian cancer and **(C)** Forest plot of EGCG in uterine cancer.

In another subgroup, GTE significantly decreased the TW in breast cancer, analyzed under fixed effect model as there was less heterogeneity among studies (Hedge’s g = -0.873, 95% CI = -1.194 to -0.552, p = 0.000, I^2^ = 42.909%, Q = 14.013, p=0.081) (**Figure 5**). Furthermore, another subgroup based on cancer type showed that EGCG significantly decreased TW in breast cancer (Hedge’s g = -2.963, 95% CI = -4.530 to -1.396, p = 0.000, I2 = 81.797%, Q = 27.467, p=0.000), ovarian cancer (Hedge’s g = -4.703, 95% CI = -7.275 to -2.132, p = 0.000, I2 = 89.598%, Q = 38.453, p=0.000), and uterine cancer (Hedge’s g = -3.742, 95% CI = -6.673 to -0.811, p = 0.012, I^2^ = 88.743%, Q = 17.767, p=0.000) (**Figures 6A-C**).

#### Adverse effects

No included studies consistently reported adverse effects following GTE, GTP, or EGCG administration. Liver function parameters were not assessed, though some studies noted no overt toxicity (e.g., (14, 17).

#### Publication bias

We conducted a qualitative assessment of publication bias based on funnel plot asymmetry and a quantitative analysis using Egger’s intercept test. While most parameters showed no bias, we applied the trim and fill method to provide unbiased estimates where bias was detected (**Supplementary Figures S1**-**S5**).

#### Sensitivity analysis

Sensitivity analysis involved systematically excluding one study at a time, revealing that no single study had sufficient impact to alter the overall conclusion (data not shown).

## Discussion

Through this meta-analysis, we evaluated the effects of GTE, GTP, and EGCG on TV and TW in preclinical models of female hormone-dependent cancers, including breast, ovarian, and uterine cancers. Preclinical models are indispensable for advancing the therapeutic development of green tea-based products in female hormone-dependent cancers, particularly when clinical data is scarce. They provide insights into efficacy, mechanisms, and dosing, all of which are essential for laying the groundwork for human trials. The pooled results demonstrated significant anti-tumor effects of these tea-based products, suggesting their potential as adjuncts in managing hormone-dependent cancers. However, we observed differential effects of GTE, GTP and EGCG on the cancer types, driven by molecular pathways and hormone receptor status, which suggest that their efficacy may vary based on the type of female hormone-dependent cancers. GTE reduces TV and TW in breast and ovarian cancers, GTP is effective in reducing TW in breast cancer, while EGCG reduces TV and TW in breast and ovarian cancers but does not significantly affect uterine cancer.

TV represents the size, shape, and overall burden of the tumor, indicating how much space it occupies within a specific anatomical region. TV assessment reflects tumor growth patterns, invasiveness, and the effectiveness of therapies (39). Our meta-analysis of 20 studies, which collectively investigated the effects of GTE, GTP, and EGCG on TV in rodent models of breast, ovarian, and uterine cancers, revealed a significant reduction in TV with these treatments (Hedge’s g = -2.332, 95% CI = -3.067 to -1.596, p = 0.000). This suggests a significant anti-tumor activity of green tea-based products in preclinical settings. The significant decrease in TV across multiple studies is promising, particularly as it spans three different cancer types, and all of which share hormonal dependence as a key driver of tumor progression.

The subgroup analysis showed that both GTE and EGCG individually exert significant reductions in TV. GTE was effective in reducing TV for breast and ovarian cancers (Hedge’s g = -1.766, p = 0.010), while EGCG showed a stronger effect across all three cancer types (Hedge’s g = -2.061, p = 0.000). These findings highlight EGCG as the most potent compound among the studied tea-based products, in line with previous research that attributes its anti-cancer properties to its ability to modulate multiple signaling pathways, including those involved in cell cycle regulation and apoptosis (40–42).

However, when considering specific cancers, the results showed some variability. For breast cancer, GTE and GTP significantly reduced TV, with GTE showing a moderate effect (Hedge’s g = -1.073, p = 0.000), and GTP showing a strong reduction (Hedge’s g = -4.281, p = 0.014). EGCG showed less consistent efficacy in breast cancer, with no significant reduction in TV (Hedge’s g = -0.806, p = 0.074), particularly in studies using triple negative cells (MDA-MB-231), indicating that its effects might vary depending on the cancer type or the experimental conditions. The lack of significance for EGCG in breast cancer warrants further investigation, as it could suggest dose-dependency or the influence of other factors such as the method of administration or the specific breast cancer subtype.

For ovarian cancer, EGCG caused a striking reduction in TV (Hedge’s g = -5.009, p = 0.000), emphasizing its efficacy in this type of cancer. On the contrary, EGCG did not significantly reduce TV in uterine cancer (Hedge’s g = -0.959, p = 0.269). This discrepancy may be due to ovarian cancer’s reliance on PI3K/AKT/mTOR and angiogenesis pathways, which EGCG effectively targets via anti-angiogenic and antioxidant effects (16, 43), compared to uterine cancer’s dependence on PTEN/PIK3CA mutations (44). Since EGCG reduced ovarian cancer, despite its typically aggressive nature compared to uterine cancer, is intriguing. Ovarian cancer is more reliant on PI3K/AKT/mTOR pathways and angiogenesis (43), making it more responsive to EGCG’s anti-angiogenic and antioxidant effects, while uterine cancer, driven more by hormonal factors and PTEN/PIK3CA mutations (44), may be less impacted. This suggests EGCG preferentially targets mechanisms underlying the growth of ovarian cancer, warranting further studies. Additionally, the animal models used in these studies may not accurately mimic the aggressive stage of ovarian cancer observed in humans, as they were not orthotopic models.

TW is a quantitative measure of tumor burden that represents changes in tumor mass, accounting for factors like cell density, necrosis, and vascularization. It also reflects the composition and biological characteristics of tumors (39, 45). The analysis of 16 studies examining the impact of GTE, GTP, and EGCG on TW in female hormone-dependent cancers revealed a significant inhibitory effect on TW across the included studies (Hedge’s g = -2.105, p = 0.000). Subgroup analyses showed that EGCG had a significant effect in reducing TW in all three cancer types with a pooled effect size of Hedge’s g = -2.885 (p = 0.000). The reductions in TW for ovarian and uterine cancers were particularly strong than its effect on breast cancer. In the context of the effects of EGCG on ovarian and uterine cancers, we observed a discrepancy: EGCG reduced TW in both cancer types, but only inhibited tumor volume TV in ovarian cancer, not in uterine cancer. Measurements of TV and TW in preclinical models can be influenced by the in tumor characteristics, and inaccuracies in measurement techniques (45). This discrepancy may reflect differences in tumor vascularity or necrosis, with EGCG potentially affecting tumor mass more than volume in uterine cancer. Additionally, variations in the stage of tumor growth and a lack of standardization in reporting can further complicate the interpretation of these measurements. Thus, this meta-analysis informs the field about the need for additional research to clarify whether EGCG has differing effects on ovarian and uterine cancers.

GTE was also found to significantly reduce TW in breast cancer (Hedge’s g = -0.873, p = 0.000), with relatively low heterogeneity (I^2^ = 42.909%), indicating consistent findings across the studies. The smaller effect size for GTE compared to EGCG may suggest that EGCG, the major catechin in green tea (46), has a relatively more potent anti-cancer effect, likely due to its greater bioavailability and ability to penetrate tissues more effectively.

Qualitative and quantitative assessments of publication bias, using funnel plots and Egger’s intercept test, indicated that most parameters were free from significant bias. However, for a few studies showing asymmetric funnel plots, we applied the trim-and-fill method to provide unbiased estimates. The use of this corrective approach supports the reliability of the findings, even in cases where potential publication bias was detected.

The strengths of our study lie in its comprehensive analysis of preclinical research on green tea compounds - GTE, GTP, and EGCG - in female hormone-dependent cancers, demonstrating significant reductions in tumor burden. By including a broad range of studies and conducting detailed subgroup analyses, this meta-analysis provides key insights into the efficacy of these compounds across different cancer types. We observed that tumor models relied on xenotransplantation, and not orthotopic models, where tumors are implanted in their tissue of origin. Orthotopic models offer advantages: they better mimic the tumor microenvironment, allow for accurate assessment of tumor growth and invasion, and enable the study of tumor-host interactions and metastasis, providing insights that closely resemble clinical condition (47). We also noted significant heterogeneity (I^2^ > 80% in several subgroups) among studies, which serve to inform this field of research. This heterogeneity could arise from differences in animal models, modes of delivery (oral, intravenous, etc.), dosing regimens, or variations in tumor induction methods. The high heterogeneity emphasizes the need for standardized experimental designs in future preclinical studies. We also observed that while GTE and GTP consistently reduced tumor burden, EGCG’s effects were more variable. This variability may be partly due to the inclusion of triple-negative breast cancers (MDA-MB-231), which are not strictly hormone-dependent, unlike ER+/PR+ cancer lines (MCF - 7), suggesting subtype-specific responses. Furthermore, there was variation in the effect in terms of tumor growth likely stemming from variations in tumor characteristics, inaccuracies in measurement techniques, and lack of standardization. Despite this variability, the overall conclusions remained consistent, as demonstrated by the sensitivity analysis, which indicated that no single study had significant influence on the results. There is also limited information on long-term safety and toxicity of these products. In particular, no included studies in this meta-analysis, which focused on xenograft and PDX models of hormone-dependent cancers, systematically assessed liver function parameters. However, one preclinical study reported that high doses of EGCG caused mild liver injury in mice, which was significantly augmented by lipopolysaccharide, while limited GT consumption showed no significant adverse liver effects over a short term (48). High-dose GTE has been linked to acute liver failure in humans (49, 50). Future studies using xenograft and orthotopic models should assess liver function to clarify the safety profile of these compounds.

## Conclusion

Overall, this meta-analysis demonstrates that GTE, GTP, and EGCG have significant anti-tumor effects in preclinical models of female hormone-dependent cancers. However, there are differential effects of these tea-based products across tumor types: GTE reduces TV and TW in breast and ovarian cancers, GTP is effective in reducing TW in breast cancer, and EGCG lowers TV and TW in breast and ovarian cancers but has limited impact on uterine cancer. These differences may reflect subtype-specific responses, with GTE and GTP showing broader efficacy across hormone-responsive cancers, while EGCG’s effects are more pronounced in cancers reliant on angiogenesis and PI3K/AKT/mTOR pathways. These findings underscore the need for targeted clinical trials to explore the specific benefits of each tea-based products analyzed in this meta-analysis for different cancer types, with a focus on hormone receptor status and molecular pathways, and evaluate their safety, efficacy, and optimal dosing.

## Data Availability

The raw data supporting the conclusions of this article will be made available by the authors, without undue reservation.

## Supplementary material

Supplementary Figure 1 Risk of bias analyses were conducted using funnel plots for TV: **(A)** the pooled group of GTE, GTP, and EGCG in female hormone-dependent cancers; **(B)** the subgroup of GTE in female hormone-dependent cancers; and **(C)** the subgroup of EGCG in female hormone-dependent cancers.

Supplementary Figure 2 Risk of bias analyses were conducted using funnel plots for TV: **(A)** the subgroup of GTE in breast cancer; **(B)** the subgroup of GTP in breast cancer; and **(C)** the subgroup of EGCG in breast cancer.

Supplementary Figure 3 Risk of bias analyses were conducted using funnel plots for TV: **(A)** the subgroup of EGCG in ovarian cancer; **(B)** the subgroup of EGCG in uterine cancer.

Supplementary Figure 4 Risk of bias analyses were conducted using funnel plots for TW: **(A)** the pooled group of GTE, GTP, and EGCG in female hormone-dependent cancers; and **(C)** the subgroup of EGCG in female hormone-dependent cancers.

Supplementary Figure 5 Risk of bias analyses were conducted using funnel plots for TW: **(A)** the subgroup of GTE in breast cancer; **(B)** the subgroup of EGCG in breast cancer; **(C)** the subgroup of EGCG in ovarian cancer; and **(D)** the subgroup of EGCG in uterine cancer.
