# Supplementary figures and images for: A systematic review and meta-analysis of the effects of green tea extracts and polyphenols in female hormone-dependent cancers for benefit-risk evaluation
Source: Front Oncol. 2025 Sep 22;15:1579470. doi: 10.3389/fonc.2025.1579470 (PMC12497591; doi:10.3389/fonc.2025.1579470)

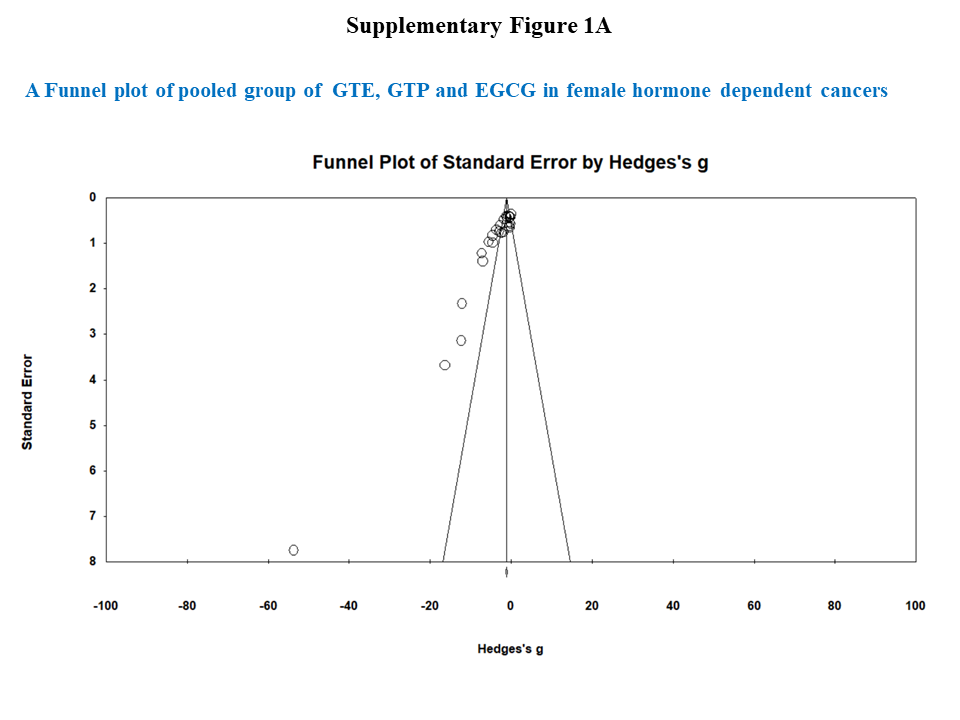

Supplement: Supplementary Figure 1 — Risk of bias analyses were conducted using funnel plots for TV: (A) the pooled group of GTE, GTP, and EGCG in female hormone-dependent cancers; (B) the subgroup of GTE in female hormone-dependent cancers; and (C) the subgroup of EGCG in female hormone-dependent cancers. [file Image1.tif]

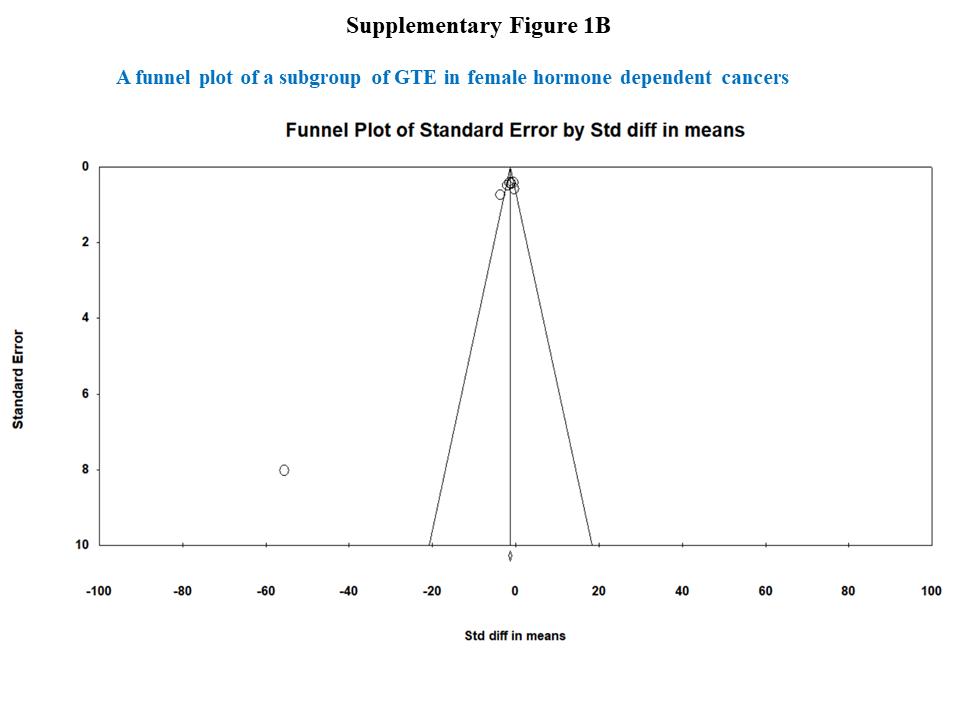

Supplement: Supplementary Figure 2 — Risk of bias analyses were conducted using funnel plots for TV: (A) the subgroup of GTE in breast cancer; (B) the subgroup of GTP in breast cancer; and (C) the subgroup of EGCG in breast cancer. [file Image2.tif]

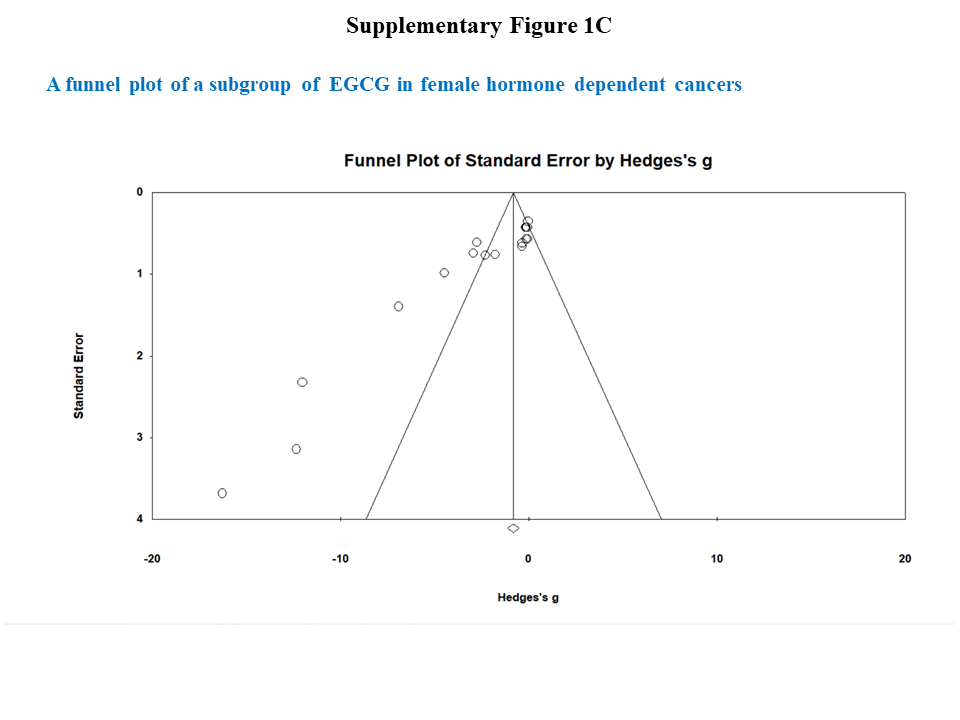

Supplement: Supplementary Figure 3 — Risk of bias analyses were conducted using funnel plots for TV: (A) the subgroup of EGCG in ovarian cancer; (B) the subgroup of EGCG in uterine cancer. [file Image3.tif]

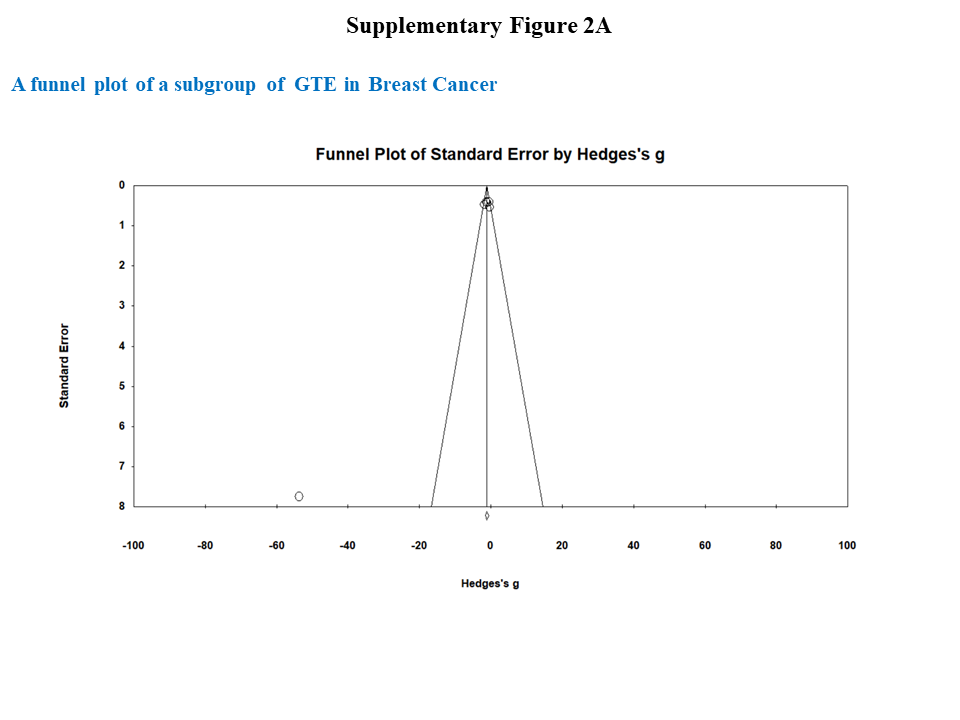

Supplement: Supplementary Figure 4 — Risk of bias analyses were conducted using funnel plots for TW: (A) the pooled group of GTE, GTP, and EGCG in female hormone-dependent cancers; and (C) the subgroup of EGCG in female hormone-dependent cancers. [file Image4.tif]

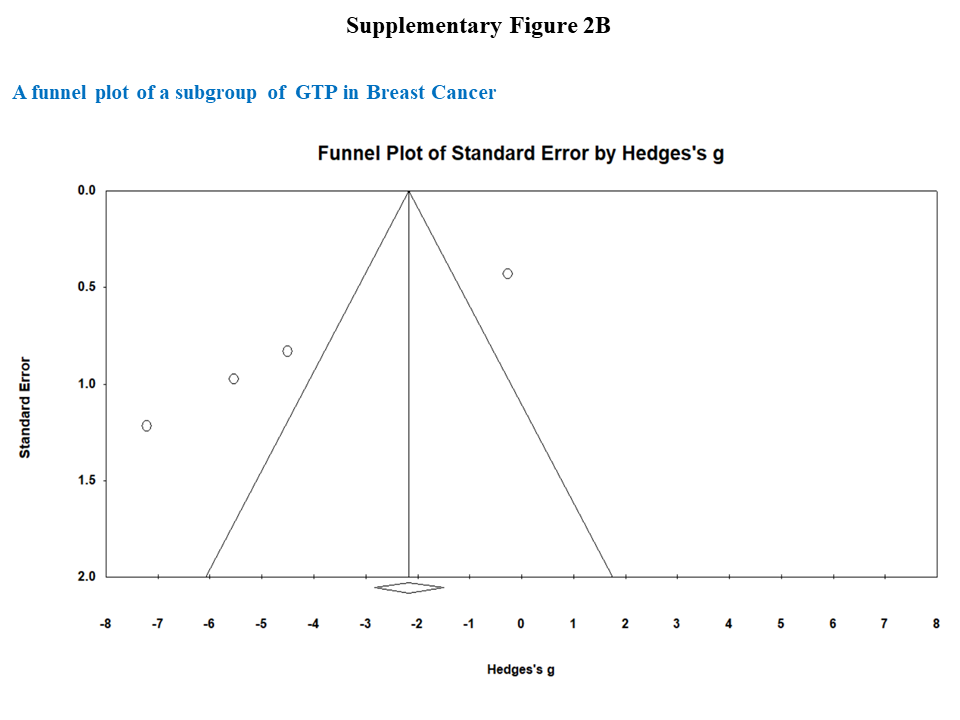

Supplement: Supplementary Figure 5 — Risk of bias analyses were conducted using funnel plots for TW: (A) the subgroup of GTE in breast cancer; (B) the subgroup of EGCG in breast cancer; (C) the subgroup of EGCG in ovarian cancer; and (D) the subgroup of EGCG in uterine cancer. [file Image5.tif]

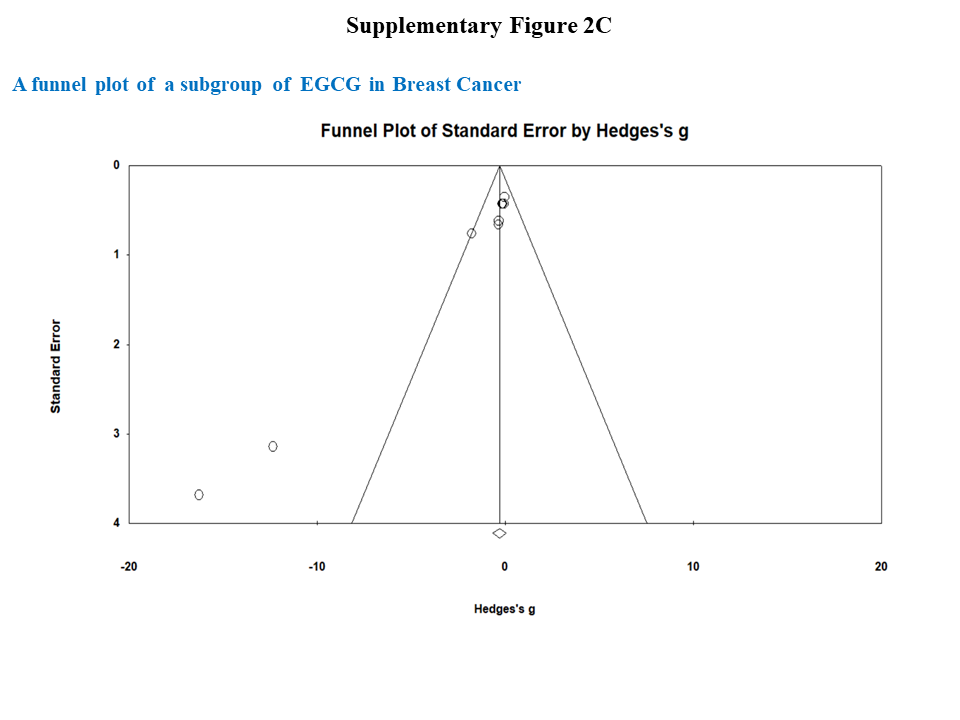

Supplement: Supplementary file 6 [file Image6.tif]

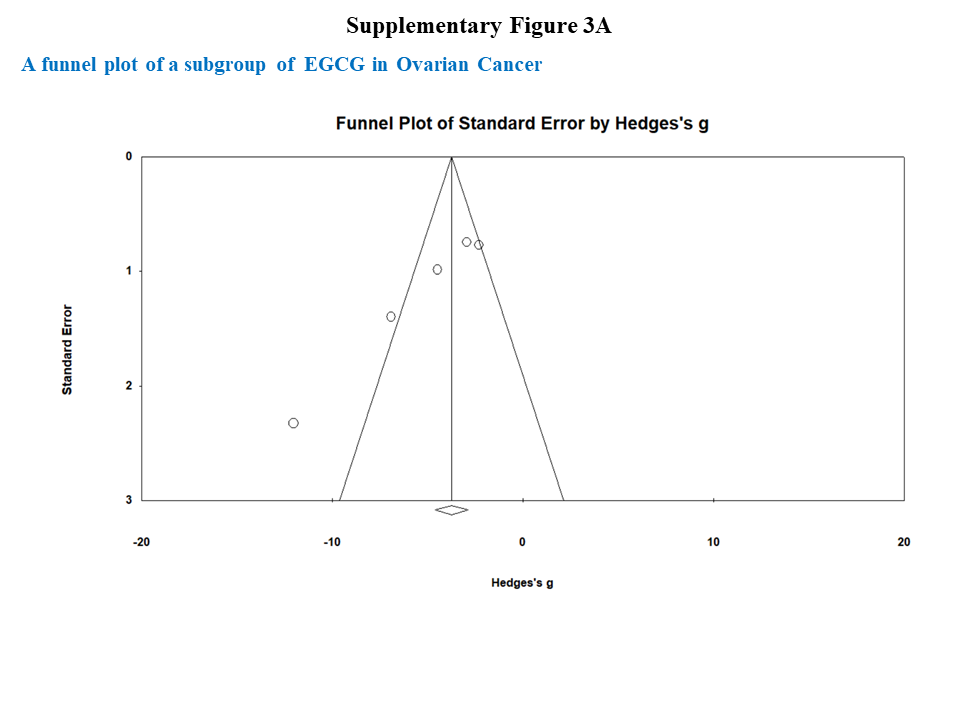

Supplement: Supplementary file 7 [file Image7.tif]

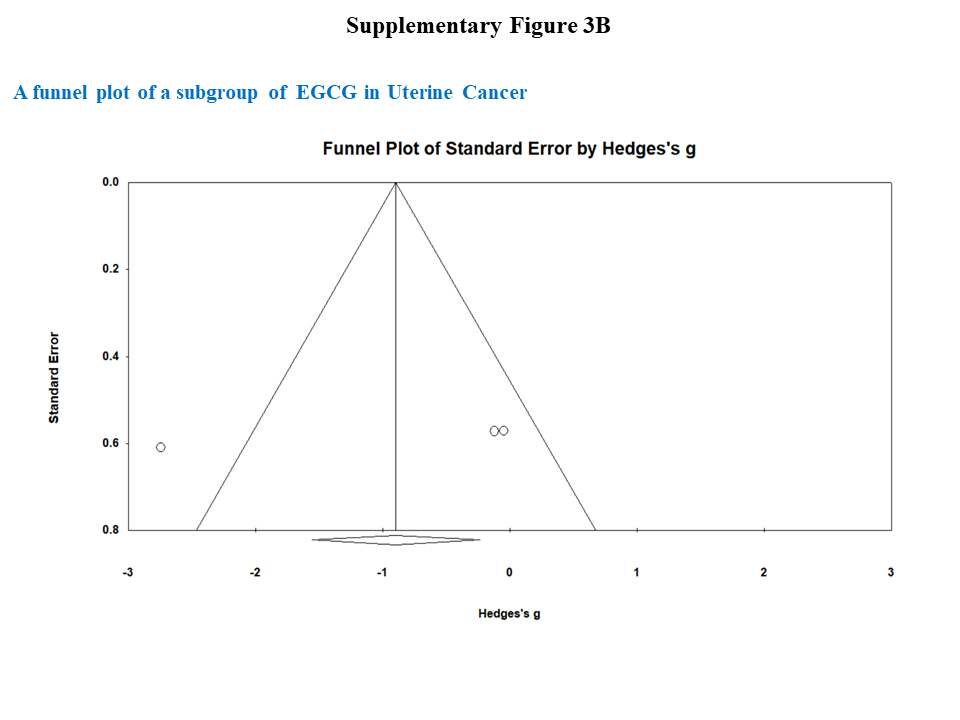

Supplement: Supplementary file 8 [file Image8.tif]

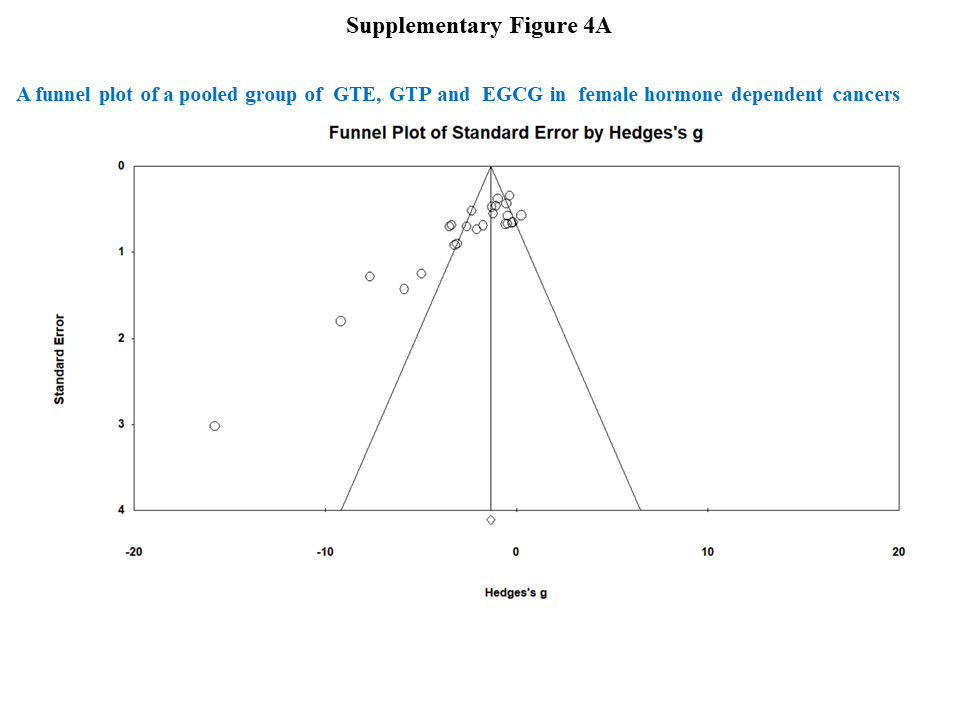

Supplement: Supplementary file 9 [file Image9.tif]

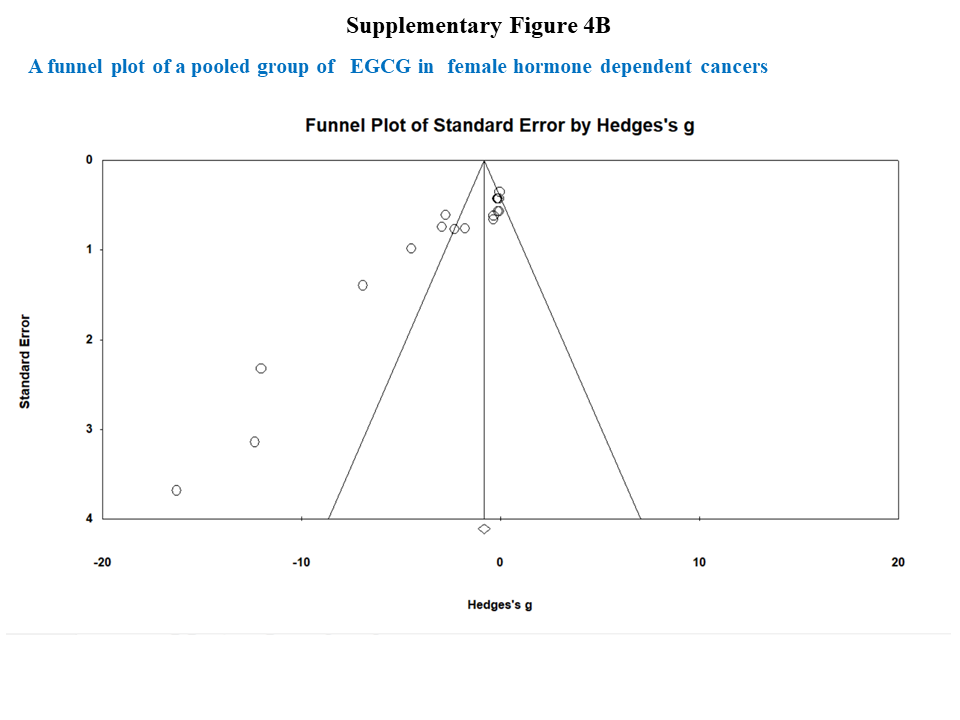

Supplement: Supplementary file 10 [file Image10.tif]

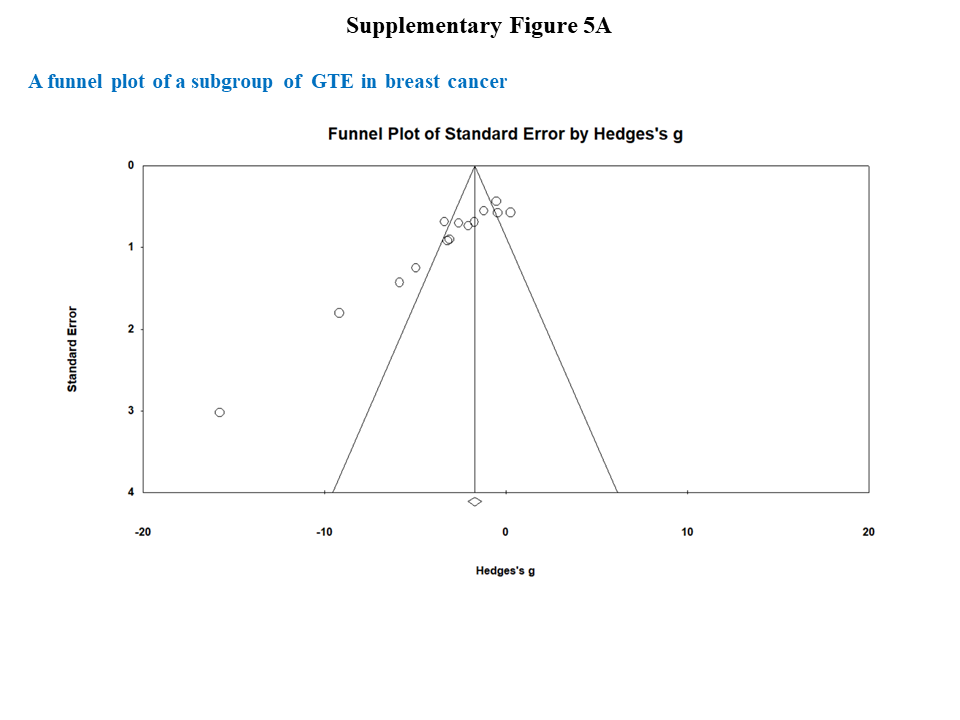

Supplement: Supplementary file 11 [file Image11.tif]

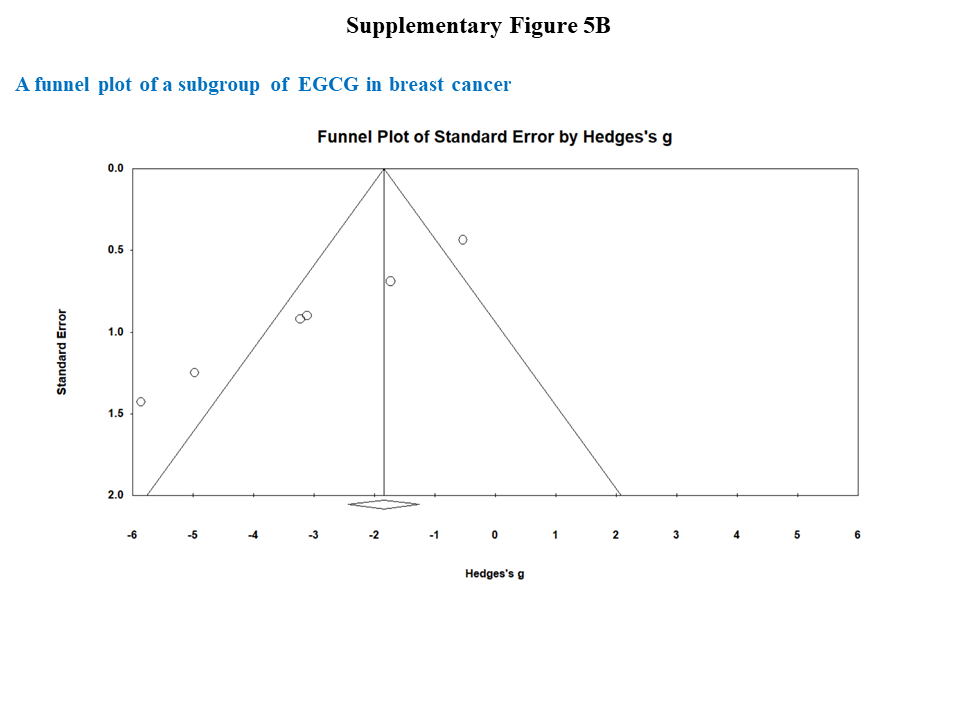

Supplement: Supplementary file 12 [file Image12.tif]

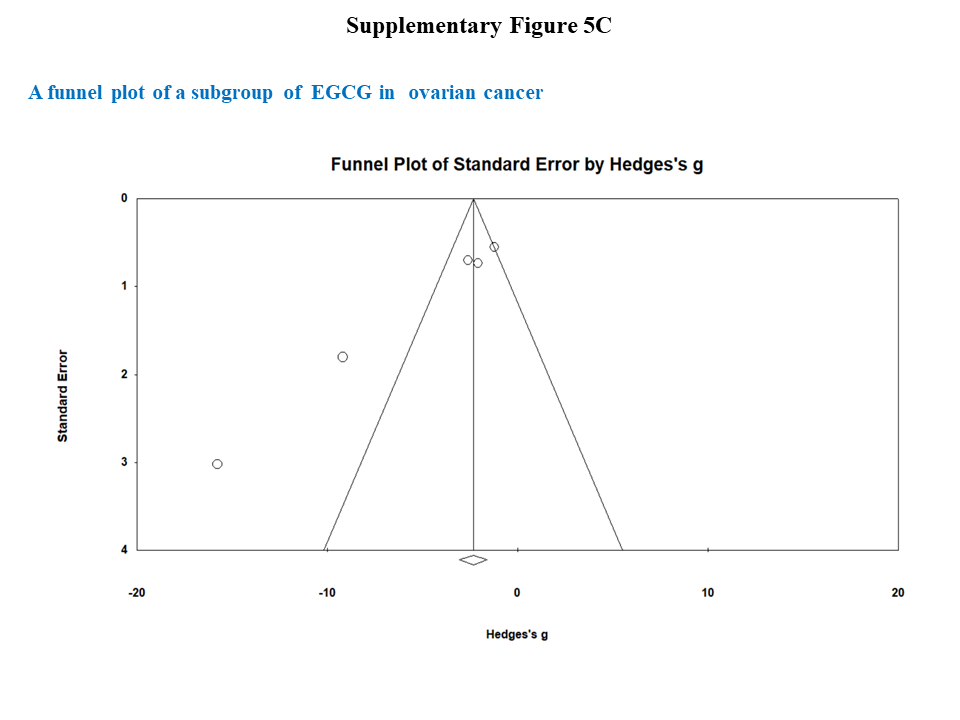

Supplement: Supplementary file 13 [file Image13.tif]

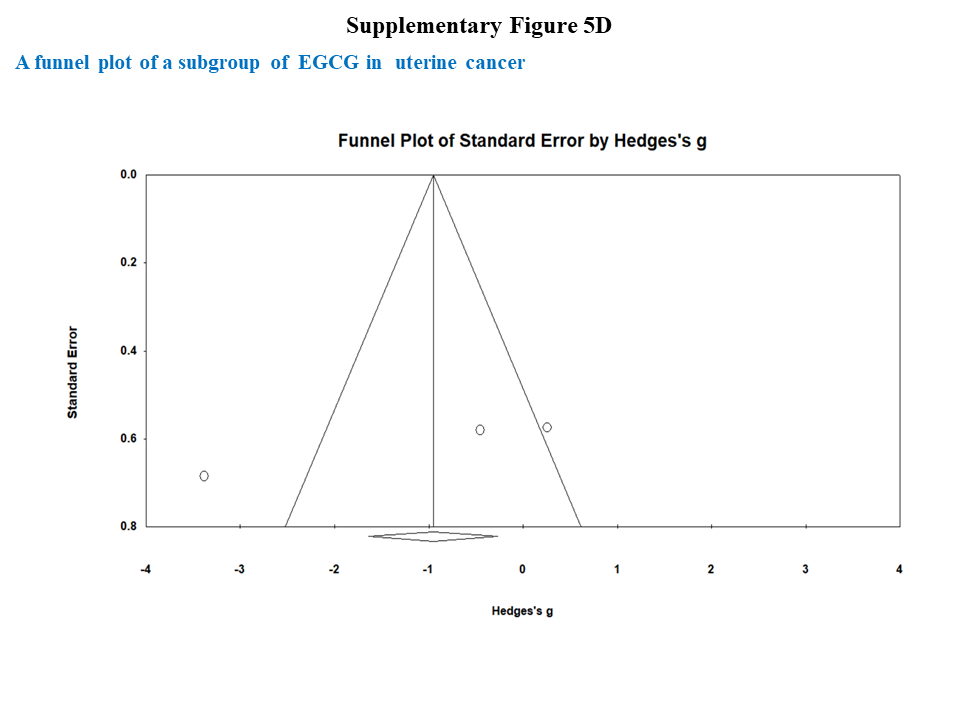

Supplement: Supplementary file 14 [file Image14.tif]
